# Increased atherosclerotic plaque in AOC3 knock-out in ApoE^−/−^ mice and characterization of AOC3 in atherosclerotic human coronary arteries

**DOI:** 10.3389/fcvm.2022.848680

**Published:** 2022-09-13

**Authors:** Anna Filip, Soraya Taleb, Rümeyza Bascetin, Mohammad Jahangiri, Matthieu Bardin, Cindy Lerognon, Bruno Fève, Patrick Lacolley, Sirpa Jalkanen, Nathalie Mercier

**Affiliations:** ^1^Université de Lorraine, Inserm, Défaillance Cardiovasculaire Aigue et Chronique (DCAC), Université de Lorraine, Lorraine, France; ^2^Inserm UMR_S970, Paris Centre de Recherche Cardiovasculaire (PARCC), Paris, France; ^3^Sorbonne Université, Inserm UMR_S938, Centre de Recherche Saint Antoine, IHU ICAN, Service d'Endocrinologie, CRMR PRISIS, APHP Hôpital Saint-Antoine, Paris, France; ^4^Medicity Laboratory, University of Turku, Turku, Finland

**Keywords:** semicarbazide-sensitive amine oxidase, vascular smooth muscle cells, atherosclerosis, inflammation, vascular adhesion protein-1, amine oxidase copper containing 3, dedifferentiation, human coronary artery

## Abstract

**Introduction:**

Amine oxidase copper containing 3 (AOC3) displays adhesion between leukocytes and endothelial cells and enzymatic functions. Given its controversial role in atherogenesis, we proposed to investigate the involvement of AOC3 in the formation of atherosclerotic plaques in ApoE^−/−^AOC3^−/−^ mice and human coronary arteries.

**Methods:**

Lesions, contractile markers, and AOC3 were studied in aortic tissues from 15- and 25-week-old mice and different stages of human coronary atherosclerotic arteries by immunohistochemistry (IHC) and/or western blot. Human VSMCs, treated or not with LJP1586, an AOC3 inhibitor, were used to measure differentiation markers by qPCR. AOC3 co-localization with specific cell markers was studied by using confocal microscopy in mice and human samples.

**Results:**

At 15 weeks old, the absence of AOC3 was associated with increased lesion size, α-SMA, and CD3 staining in the plaque independently of a cholesterol modification. At 25 weeks old, advanced plaques were larger with equivalent staining for α-SMA while CD3 increased in the media from ApoE^−/−^AOC3^−/−^ mice. At both ages, the macrophage content of the lesion was not modified. Contractile markers decreased whereas MCP-1 appeared augmented only in the 15-week-old ApoE^−/−^AOC3. AOC3 is mainly expressed by mice and human VSMC is slightly expressed by endothelium but not by macrophages.

**Conclusion:**

AOC3 knock-out increased atherosclerotic plaques at an early stage related to a VSMC dedifferentiation associated with a higher T cells recruitment in plaques explained by the MCP-1 augmentation. This suggests that AOC3 may have an important role in atherosclerosis independent of its canonical inflammatory effect. The dual role of AOC3 impacts therapeutic strategies using pharmacological regulators of SSAO activity.

## Introduction

Oxidative stress, inflammation, and vascular smooth muscle cell (VSMC) phenotype switching are important events driving atherosclerosis. Indeed, atherosclerosis is a chronic inflammatory disease of large- and medium-sized arteries characterized by intimal thickening that develops by an early accumulation of oxidative low-density lipoprotein (oxLDL) particles that become pro-inflammatory and immunogenic followed by an infiltration of monocytes-macrophages and lymphocytes into the plaque (1). In humans, some VSMC already located in the intima can switch their phenotype and proliferate directly in this layer. However, there is no VSMC in the intima of normal rodent vessels. Thus, VSMC switches their phenotype to proliferate and migrate to the intima where they synthesize the fibrous cap to stabilize the plaque. They can also become macrophage-like cells which promote inflammation (2).

Amine oxidase copper containing 3 (AOC3) is an enzyme that belongs to the semicarbazide-sensitive amine oxidase (SSAO) family also known as vascular adhesion protein-1 (VAP-1). It converts primary amines into corresponding aldehydes, H_2_0_2_, and ammonium. Expressed on lymphatic's endothelium under inflammation, its inhibition diminishes leukocyte migration and inflammatory in various pathologies (3–5). AOC3 seems an interesting potential target in the treatment of atherosclerosis. Also expressed in arterial VSMC, its expression and activity are increased upon their late differentiation (6) and could be involved in the organization of the extracellular matrix (7–10). AOC3 accelerates terminal differentiation (11, 12) and participates in cardiovascular calcification (13). Secreted in the serum by VSMC (7, 14), AOC3 is an independent marker of atherosclerosis (15–17) and can extend to coronary artery disease (18). Inhibition of AOC3 in limiting atherosclerosis is controversial, showing sometimes either a reduction (18, 19), an increase (20) in atheromatous plaques, or a stabilization (21) through a phenotypic switch of VSMC. Previous works (8, 9) have shown a 40% SCZ-induced lysyl oxidase inhibition, an enzyme highly expressed by VSMC with various implications in the cardiovascular system (22).

This work presented here aims to assess the consequences of a total deletion of AOC3 on the development of atheromatous plaques in ApoE^−/−^ mice under a normal diet and to preclude the possible inhibitory off-target effects as well as to investigate the influence of the HF/HC diet *per se*. Our results show an increase in plaque surface from 15 to 25 weeks of age accompanied by dedifferentiation of VSMC, an increase in infiltrated CD3^+^ cells in the plaques, and the media is associated with an increased MCP-1 expression in ApoE^−/−^AOC3^−/−^ mice when compared to ApoE^−/−^ mice. AOC3 co-localizes with VSMC in the media and the plaque in mice and humans and slightly with endothelial cells. VAP-1 implication in VSMC differentiation seems more important than expected and should be considered with caution with regard to potential therapeutic targeting.

## Materials and methods

### Patients

Human healthy and diseased coronary arterial walls were collected from the Inserm human CV biobank (BB-0033-00029, U 1148, X. Bichat Hospital, Paris), included in the European network BBMRI-ERIC, in accordance with the French regular and ethical rules (BioMedicine Agency convention DC2018-3141) and the principles of the declaration of Helsinki. Approval was obtained from the French Biomedical Agency (ABM, PFS09-007& PFS17-002) and the Institutional Ethical Review Board (SC09-09-66). Tissues were obtained from deceased organ donors for kidney and/or hepatic transplantation in the absence of therapeutic uses for the heart. When collected, coronary arteries were longitudinally opened and macroscopically analyzed by a trained vascular surgeon (Michel JB.) to classify them into healthy, fatty streak, and atheroma patients. Tissues were fixed with 4% (w/v) buffered formaldehyde solution prepared by depolymerization of paraformaldehyde and embedded in paraffin. Serial 5 μm thick sections were performed. The general structure of human coronary artery sections was observed after an elastic Van Gieson staining kit (Merck, 1.15974, Darmstadt, Germany) coloration and was used in addition to CD68+ foam cell staining to classify each quantified area according to the American Heart Association (23, 24).

### Animals

All experiments were performed in accordance with national animal care guidelines and were approved by a local ethics committee and the Ministry of National Education, Higher Education, and Research (Approval number: 01637.02). Mice were housed under controlled temperature and light cycle conditions with a normal diet (SDS, CRM E 801730, Argenteuil, France) and water available *ad libitum*. ApoE^−/−^AOC3^−/−^ double knockout mice were generated as follows: the AOC3^−/−^ knockout mice in a C57BL6/J background were established as described previously (25). In brief, gene targeting technics were used to disrupt the mouse AOC3 gene by replacing a portion of its first exon with a neomycin-resistance (Neor) cassette. Mice homozygous for the null mutation were produced and this mutation was maintained in a pure C57 background. AOC3^−/−^ mice were crossbred with ApoE^−/−^ knockout mice (Apoe tm1Unc/J) in a C57BL6/J background from the Jackson Laboratory (Charles River, L'Arbresle, France) to obtain double knockout mice (ApoE^−/−^AOC3^−/−^). Mice were put to death at 11, 15, 25, and 70 weeks of age with 3.5% of isoflurane in oxygen, and the blood was collected to get the serum. Hearts were fixed in 4% paraformaldehyde (PAF) and embedded in OCT, and frozen and kept at −80°C. Thoracic aortas were fixed in 4% formaldehyde and abdominal aortas were kept frozen at −80°C until use.

### Size and composition of the atherosclerotic lesion by histology and immunohistochemistry

Thoracic aortas from mice were opened longitudinally and stained with oil red O (Merck, O0625, Darmstadt, Germany). Serial 10 μm transversal sections of the aortic sinus were performed and kept at the beginning of the appearance of the tricuspid valves. Lipids were detected using oil red O staining, and elastic and global structure with a Van Gieson staining kit (Merck, 1.15974, Darmstadt, Germany). For mouse IHC, MOMA-2, CD3, alpha-smooth muscle actin (α-SMA), AOC3, and muscle myosin heavy chain (SM-MHC) were detected in consecutive aortic sinus sections. For human IHC, CD68, AOC3, Von Willebrand factor (VWF), and α-SMA were detected on consecutive coronary sections. In both cases, corresponding secondary antibodies based on the standard avidin-biotin-peroxidase complex method and 3,3′-diaminobenzidine (Vector Labs, Newark, USA) as a substrate were used to localize the primary antibodies. The preparations were mounted with Permount (Merck, Darmstadt, Germany). All pictures under the light microscope (Nikon DIAPHOT 300, Tokyo, Japan) were recorded using the Nikon digital camera (SIGHT DS-Fi1, Tokyo, Japan) and quantified with NIS-BR 3.2 software. See Supplementary Table 1 for details on antibodies.

### VSMC differentiation markers in abdominal aortas by western blot

Western blot membranes performed with individual lysates from abdominal mouse aortas were blotted with the following primary antibodies directed against anti-smooth muscle myosin heavy chain SM-MHC anti-alpha smooth muscle actin (α–SMA), anti h-caldesmon, anti-smoothelin, and GAPDH (see Supplementary material and methods section).

### Expression of differentiation markers in human VSMC by real-time PCR

Human Aortic VSMC (CC-2571) were plated in six well-plates and cultured until 70% confluence in growth medium containing SmBMTM Basal Medium (CC-3181) and SmGMTM-2 SingleQuotsTM supplements (CC-4149), including 5% fetal bovine serum (FBS). All products are from Lonza, Verviers, Belgium. Then, cells were cultured in 0.1% FBS for 48 h and either treated or not with LJP1586 at 1 μM (an AOC3 inhibitor) for the next 48 h. RNAs were extracted with the RNeasy kit (Qiagen, ID: 74104, Courtaboeuf Cedex, France) according to the manufacturer's instructions. RT-qPCR was carried out as mentioned in Supplementary material and methods section. The primer details can be found in Supplementary Table 2. The gene encoding RPS29 was used as a standard. The 2^−ΔΔCt^ methods were used for calculation.

### Statistical analysis

The results were presented as individual dots for one animal and the mean value ± SD was given for each group. Because the normality was not verified, a non-parametric Mann–Whitney test was used to assess if the difference between ApoE^−/−^AOC3^−/−^ and ApoE^−/−^ at corresponding ages was significant. For human samples, a Kruskal–Wallis test was performed to compare healthy, fatty streak, and atheroma groups. When significant, a Mann-Witney test was done to verify the level of significance between the two groups; *p*-values < 0.05 were considered significant. The number of animals or human samples is given in the figure legends.

## Results

### Serum AOC3 concentration and tissue expression during the development of mouse atherosclerosis

The AOC3 protein concentration measured by ELISA in sera of ApoE^−/−^ mice was significantly increased by 20-folds in 70-week-old mice compared to 11-week-old or compared to 70-week-old wild-type (WT) mice (Supplementary Figures 1A,B). As expected, AOC3 protein was not detected in the serum of AOC3^−/−^ mice deficient for the AOC3 gene that was used as a negative control. AOC3 protein expression was gradually increased in the abdominal aorta in ApoE^−/−^ mice aging by western blot (Supplementary Figures 1C,D) to reach a statistical difference at 70 weeks. In the aortic sinus, AOC3 is strongly expressed in the media (57.8% of the media) at 15 weeks old but significantly decreased at 25 weeks old (37.2% of the media). In the lesion, no difference was found between 15- and 25-week-old mice but a high heterogeneity could be observed (Supplementary Figures 1E,F). These results show soluble and tissular AOC3 increases with atherosclerosis severity and could also be a marker of the severity of the disease in ApoE^−/−^ mice.

### Lesion size in thoracic aorta and composition of the lesion in aortic sinus

**Figure 1:**
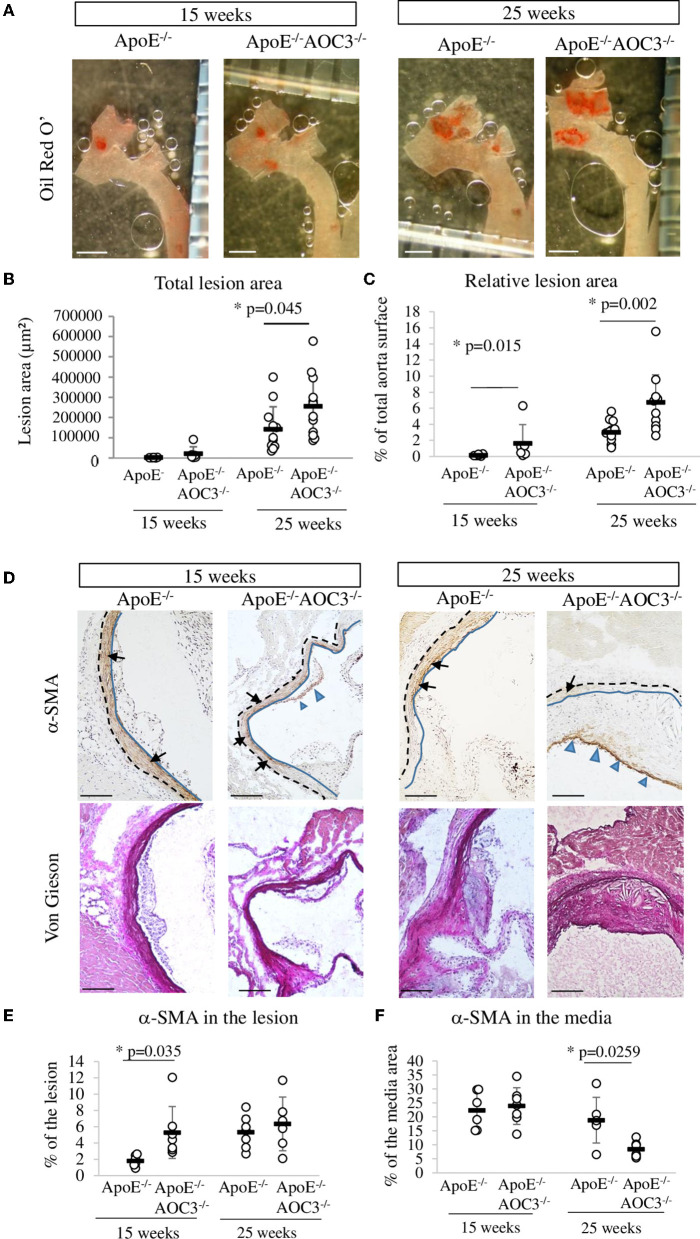
Lesion surface in the thoracic aorta and α-SMA in the aortic sinus. The total lesion area was determined by measuring the red staining made by Oil Red O' staining in the aorta open in ApoE^−/−^ and ApoE^−/−^AOC3^−/−^ female mice at 15 and 25 weeks of age. **(A)** Representative pictures of a partial thoracic aorta (part of ascending, arch, and descending aorta) stained by Oil Red O' (the bar scale represents 1 mm). **(B)** Plaque surface quantification is expressed as μm^2^ or **(C)** as a percentage of the total surface of the thoracic aorta. *N* = 6 per genotype of 15-week-old mice and *n* = 12 of 25-week-old mice. **(D)** Alpha actin (α-SMA) was detected by immunohistochemistry in the aortic sinus. Representative micrographs of mouse aortic sinus in each group. Blue-headed arrows and black arrows indicate, respectively, the brown positive staining in the media and the lesion. The blue line limits the media and intima whereas the black dotted line separates the media from the adventitia. Van Gieson staining of the corresponding slices is provided. 6<*n*<9. The bar scale measures 100 μm. **(E)** The quantification of α-SMA was determined in the lesion and is expressed as percentage of the lesion area. **(F)** The α-SMA expression was quantified in the media and the results are expressed as percentage of the media surface. Each dot stands for a single animal and the line represents the mean of the group +/- SD. *p < 0.05.

The absence of AOC3 induced a significant 1.8-fold increase in the total surface area of early to intermediate lesions in the thoracic aorta at the age of 15 weeks compared to ApoE^−/−^ mice (Figures 1A,B). Similar results were obtained when lesions were quantified in the aortic sinus at 25-week-old equivalent to advanced plaque (26) (Supplementary Figure 2). When expressed as the percentage of the aorta surface, the plaque surface was already significantly increased in 15-week-old ApoE^−/−^VAP-1^−/−^ mice compared to ApoE^−/−^ mice (Figure 1C). Total cholesterol, LDL-cholesterol, triglyceride concentrations, and body weight were not modified (Supplementary Table 3). These results were confirmed in male mice (Supplementary Figure 3). In contrast, α-the SMA signal was slightly but significantly increased in lesions (Figures 1D,E) but not in the media of 15-week-old ApoE^−/−^AOC3^−/−^ mice compared to ApoE^−/−^ mice (Figures 1D,F). At 25 weeks of age, the level of α-SMA staining was comparable in the atheroma, whereas it was decreased in the media of double knockout mice compared with ApoE deficient mice. No significant differences in total collagen were observed with Sirius red staining at both ages (data not shown).

**Figure 2:**
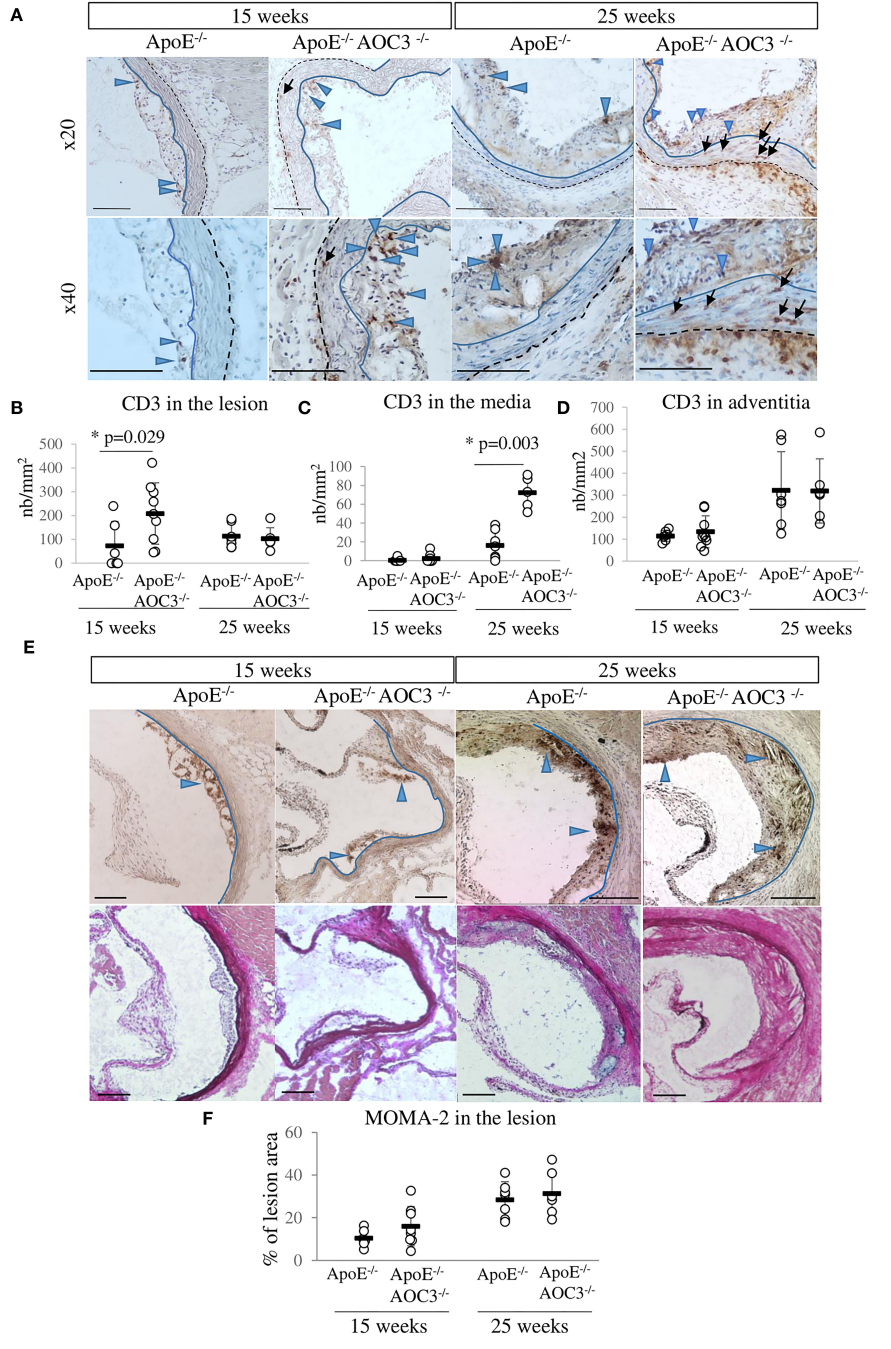
CD3^+^ T-cell and macrophage infiltrations in the aortic sinus. CD3^+^ T-cells and MOMA-2^+^ macrophages were detected by immunohistochemistry in the aortic sinus of 15- and 25-week-old ApoE^−/−^ and ApoE^−/−^AOC3^−/−^ female mice (*n* = 6–9). **(A)** Representative micrographs of mouse aortic sinus for each group. The bar scale represents 100 μm. Blue-headed arrows and black arrows indicate examples of the brown positive staining in the media and the lesion, respectively. The blue line limits the media and intima whereas the black dotted line separates the media from the adventitia. Van Gieson staining of the corresponding slices is provided. **(B)** The number of CD3^+^ T-cells was counted in the lesion area, **(C)** in the media, and **(D)** in the adventitia. The results are expressed as the number of CD3^+^ T-cells/mm^2^ of the lesion or the media. **(E)** Macrophages were detected by an anti-MOMA-2 antibody. Representative pictures from aortic sinuses of ApoE^−/−^ and ApoE^−/−^AOC3^−/−^ mice at 15 and 25 weeks of age are shown. Blue-headed arrows indicate examples of the brown positive staining in the media and the lesion. **(F)** The results are expressed as percentage of the lesion area. Each dot stands for a single animal and the line represents the mean of the group +/- SD. *p < 0.05.

Infiltration of T lymphocytes (CD3^+^) and monocytes/macrophages (MOMA-2) into the aortic sinus of ApoE^−/−^ and ApoE^−/−^AOC3^−/−^ mice at the ages of 15 and 25 weeks was detected by IHC (Figure 2). In 15-week-old mice, the number of CD3^+^ cells increased significantly in plaques in ApoE^−/−^AOC3^−/−^ compared to ApoE^−/−^ as shown in the pictures by the blue arrowheads in Figure 2A and the quantification in Figure 2B. This difference was no longer observed at 25 weeks of age.

Usually considered as an immuno-privileged area (no lymphocyte infiltration), the media layer was almost free of CD3+ cells (Figures 2A,C) in the ApoE^−/−^ mice at 15 and 25 weeks of age. That was also the case at 15 weeks of age for ApoE^−/−^AOC3^−/−^ mice. However, AOC3 knockout induced a small but significant increase in CD3+ cell infiltration into the media as shown by the black arrows in the pictures (Figure 2A) and the quantification in Figure 2C at 25 weeks of age. According to the pictures (Figure 2A), CD3+ cells were more numerous in adventitia from 15- to 25-week-old ApoE^−/−^VAP-1^−/−^ mice compared with ApoE^−/−^. Even if there was a disparity in the fields observed, the lack of VAP-1 did not modify the infiltration of CD3^+^ cells on average in adventitia in both genotypes (Figure 2D).

There were no differences in the infiltration of macrophages into the plaques in mice from both genotypes neither at 15 nor at 25 weeks of age (Figures 2E,F).

### Cytokine/chemokine profiles in the spleen and the abdominal aorta

Anti-inflammatory IL10, TGFα (Supplementary Figures 4A,B), and pro-inflammatory IFNγ TNFα (Supplementary Figures 4C,D), cytokine mRNA levels were not modified in the spleen in any groups.

**Figure 3:**
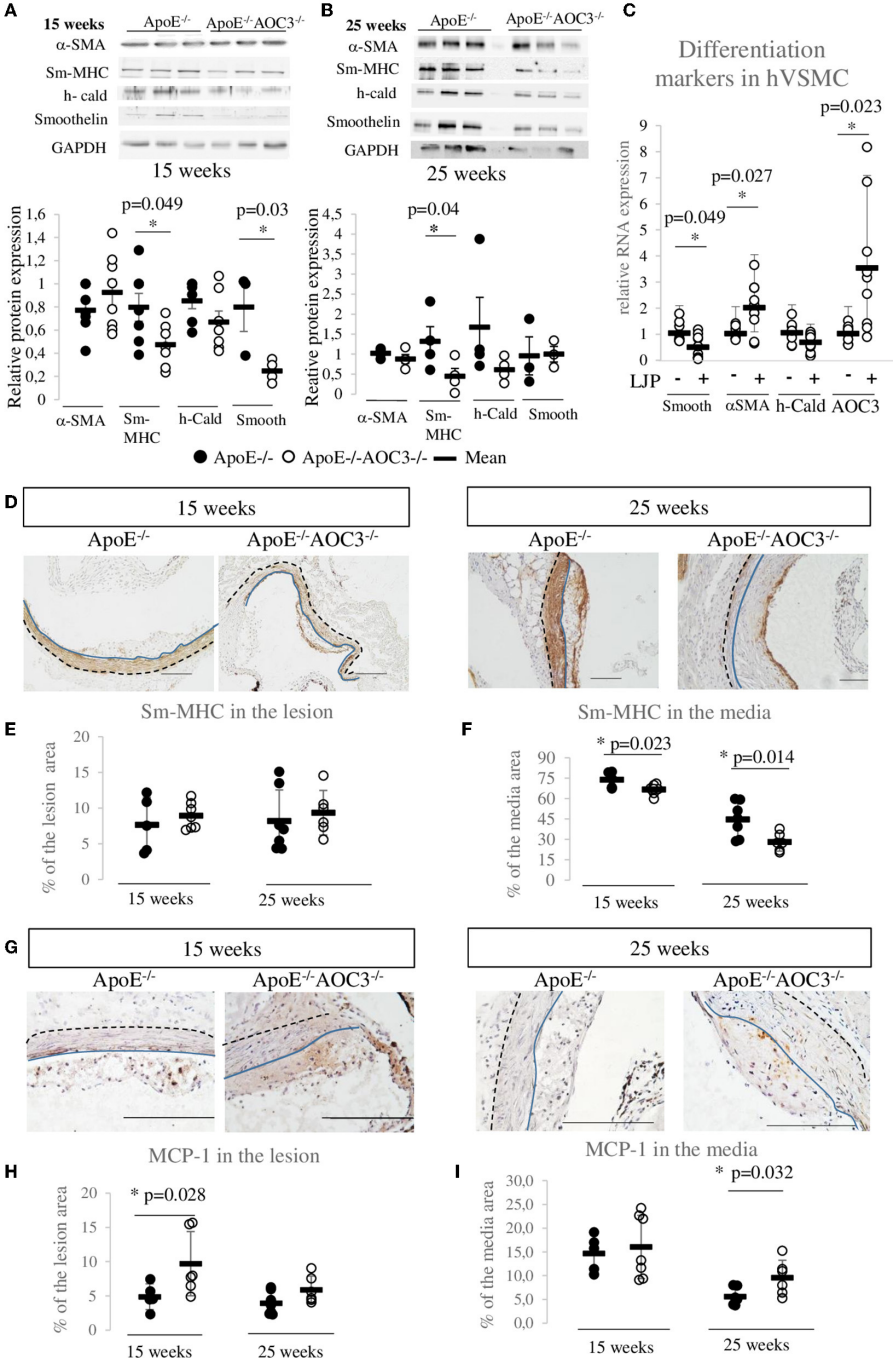
VSMC dedifferentiation and MCP1 increase induced by AOC3 knockout. VSMC differentiation markers (Sm-MHC), α-actin (α-SMA), h-caldesmon (h-Cald), and smoothelin (smooth) were quantified in abdominal aortas from ApoE^−/−^ (•) and ApoE^−/−^AOC3^−/−^ (°) female mice by Western blotting. Representative pictures and quantifications are shown for 15-week-old mice **(A)** and 25-week-old mice **(B)**. The results are given as the relative protein expression levels normalized by the GAPDH expression level. Each dot represents the result for one animal. The difference is significant (*) when *p* < 0.05. *n* = 3–4 per group in 25-week-old mice and *n* = 7–8 in 15-week-old mice. **(C)** At 70% confluence, human aortic VSMC were cultured in 0.1% fetal bovine serum and treated (+) or not (-) with LJP1586 at 1 μM (an AOC3 inhibitor) for the next 48 h. The mRNA expressions of α-SMA, h-cald, and smoothelin were measured by RT-qPCR (9<n<11). The results are normalized by RPS29 and results are given as the relative expression of non-treated cells. **(D)** Sm-MHC was also detected in the aortic sinus of 15- and 25-week-old ApoE^−/−^ and ApoE^−/−^AOC3^−/−^ female mice by immunohistochemistry (*n* = 5–7). The quantification of Sm-MHC was determined in the lesion **(E)** and the media **(F)** and is expressed as percentage of the lesion or media area. Each dot stands for a single animal and the line represents the mean of the group +/- SD. *p < 0.05. **(G)** MCP-1 was measured in the aortic sinus of 15- and 25-week-old ApoE^−/−^ and ApoE^−/−^AOC3^−/−^ female mice by immunohistochemistry (*n* = 5–7). The quantification of sm-MHC was determined in the lesion **(H)** and the media **(I)** and is expressed as percentage of the lesion or media area. Each dot stands for a single animal and the line represents the mean of the group +/- SD. *p < 0.05. Representative micrographs of mouse aortic sinus for each group. The scale bars represent 100 μm. The blue line limits the media and intima whereas the black dotted line separates the media from the adventitia.

In the abdominal aorta of ApoE^−/−^ and ApoE^−/−^AOC3^−/−^ mice, the cytokines protein array at 15 weeks of age (Supplementary Figures 4E,F) showed an interesting 1.6-fold increase in MCP-1 in ApoE^−/−^AOC3^−/−^ compared to ApoE^−/−^ mice that could participate to the increased recruitment of CD3^+^ cells in the arterial wall. This difference was not seen in 25-week-old mice (Supplementary Figures 4G,H). By IHC, MCP-1 was found augmented almost by 2-fold in the lesion at 15 weeks of age (Figures 3G,H) and in the media at 25 weeks of age in the ApoE^−/−^AOC3^−/−^ mice vs. ApoE^−/−^ aortic sinus (Figures 3G,I).

### Late differentiation markers of VSMC in the abdominal aorta and the aortic sinus

VSMC phenotype switch is a very important event in the development of atherosclerosis. At an early/intermediate stage of atherosclerosis (15-week-old mice), the level of α-SMA (VSMC lineage marker) was similar in both genotypes, while sm-MHC and smoothelin (all terminal differentiation contractile markers) were significantly decreased in ApoE^−/−^AOC3^−/−^ mice compared to ApoE^−/−^ mice by western blot (Figure 3A). At 25 weeks of age, double knockout mice still presented significantly decreased sm-MHC, an actin-myosin binding protein (Figure 3B). Expression of the sm-MHC was not different at the lesion site neither at 15- nor at 25-week-old (Figures 3D,E). The sm-MHC decrease was confirmed in the media of the aortic sinus from 15- (Figures 3D,F) and 25-week-old mice by immunohistochemistry. In ApoE-/-mice sinus, the sm-MHC expression in the media and the plaque is very similar to AOC3 (Supplementary Figures 1E,F), and it is strongly expressed in the 15-week-old mice media and decreases at 25 weeks when plaques are more developed and established. Human aortic VSMC cultured with an AOC3-enzymatic inhibitor LJP1586 presented a significantly decreased mRNA expression of smoothelin by 50% compared to untreated cells (Figure 3C). The LJP1586-induced AOC3 inhibition provokes a positive retro-control on the AOC3 mRNA expression. LJP induced a 2-fold α-SMA mRNA expression increase suggesting that VSMC has proliferated. These *in vivo* and *in vitro* results suggest that the altered contractile VSMC phenotype observed in the ApoE^−/−^AOC3^−/−^ mice aortic wall and sinus seem to be caused by the genetic deletion of VAP-1. Thus, the increased lesion development could be explained through a phenotypic switch of VSMC.

### AOC3 expression in healthy and diseased human coronary arteries

**Figure 4:**
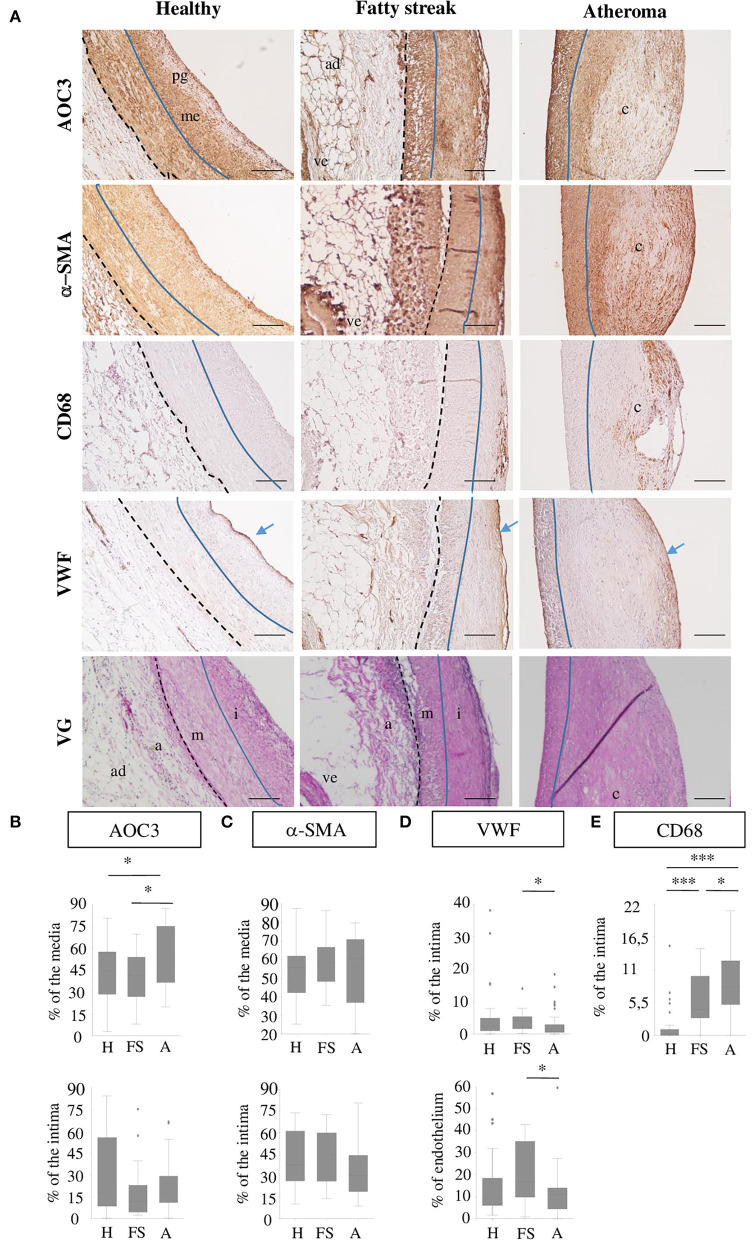
AOC3 in human coronary arteries. **(A)** Representative pictures from immunohistochemistry for AOC3, α-SMA, CD68, and VWF are shown on consecutive healthy (H), fatty streak (FS) and atheroma human carotid sections. An Elastica Van Gieson (VG) staining from consecutive sections is provided. The scale bars represent 100 μm. The blue line limits the media (m) and intima (i) that can contain proteoglycans (pg) and musculo elastic (me) layers, whereas the black dotted line separates the media from the adventitia (a) where blood vessels (ve) and adipocytes (ad) can be visible. Blue arrows show the endothelium. The signals are expressed as a percentage of the area measured (media, intima, endothelium) and the results are represented as box plots for AOC3 **(B)**, α-SMA **(C)**, VWF **(D)**, and CD68 **(E)**. Totally, 34–41 healthy areas, 18–20 fatty streaks, and 38 to 43 atheroma areas were analyzed according to the marker. *0.01<*p*<0.05; **0.001 <*p*<0.005; ***p < 0.001.

Coronary artery sections were stained by IHC for AOC3, α-SMA, CD68, and VWF and by Elastica Van Gieson staining (Figure 4A). A total of 41 healthy coronary sections of which 58.4% were from males with a mean age of 56.9 ± 8.2 years showed a limiting intimal thickening without or with very few scattered CD68+ cells indicating a limiting amount of foam cells at that stage. AOC3 is expressed in a similar pattern as α-SMA, that is, in the media, in the musculoelastic layer, and on the top of the proteoglycan layer. Even if AOC3 seems expressed similarly to VWF, it is difficult to affirm that endothelial cells express AOC3. In the 20 fatty streak (Type II-III) lesions observed (50% males; mean age 57.9 ± 10.7 years), some layers of CD68+ cells were found in the upper part of the intima, in a highly α-SMA positive area. AOC3 staining is more heterogeneous in the intima of the FS group. In total, 43 atheroma (Type IV) lesions (64.3% males; mean age 58.6 ± 6.5 years) presented a disruptive core with a poor cellularity level surrounded by layers of VSMC (α-SMA positive cells). CD68+ cells were found in intima around the core of the plaque surrounded by α-SMA and AOC3 positive area staining. When groups are compared, AOC3 significantly increased by 25.6% in media under the atheroma lesion compared to healthy media and by 38% compared to the media under FS. No difference was found in the intima neither for AOC3 (Figure 4B) nor for α-SMA (Figure 4C). VWF staining the endothelium is strongest in the FS when inflammation is high and tecruititment of immune cells is important (Figure 4D). Figure 4E shows the progressive augmentation of the CD68+ foam cells.

### AOC3 co-localization study in ApoE–/– aortic sinus and human coronary arteries

Exploration of cell-type expression of AOC3 during the development and progression of murine and human atherosclerosis was performed by co-immunofluorescent staining between AOC3 and α-SMA (Supplementary Figure 5A), CD31 (Supplementary Figure 5B), and MOMA-2 (Supplementary Figure 5C). In 15-week-old mice, the AOC3 signal either overlaps or surrounds the α-SMA signal in the media according to the plot profiles. The M1 and M2 coefficients show that 32.1% of AOC3 expressing cells also express α-SMA and 12.3% of the α-SMA expressing cells also express AOC3. At 25 weeks of age, the media did not show any co-localization between AOC3 and α-SMA. The endothelial cells show a slight co-localization between CD31 and AOC3 probably both expressed at the membrane according to the plot profile at 15 weeks old. At 25 weeks old, no significant co-localization was visible (Supplementary Figure 5B). In any case, the AOC3 signal was detected in macrophages (MOMA+ cells) (Supplementary Figure 5C), indicating that AOC3 is mostly expressed by VSMC and slightly by endothelial cells. In all pictures observed, an AOC3 signal was detected in the adipocytes and blood vessels found in the adventitia.

In human coronary arteries (Supplementary Figure 6), the plot profiles within the intima and media of healthy (Supplementary Figure 6A), fatty streak (Supplementary Figure 6B), and atheroma (Supplementary Figure 6C) tissues show either an overlap of AOC3 signal with α-SMA signal or an AOC3 signal surrounding the α-SMA signal. These data show that AOC3 is localized in cells or at the membrane of cells expressing α-SMA. M1 and M2 show that at least 50% of α-SMA co-localize with AOC3 within the selected regions within the media and the intima in healthy, fatty streak and atheroma grade, except in the core of the plaque (green square) where neither α-SMA nor AOC3 was detected. These results confirm that AOC3 is at least expressed by human VSMC in healthy and atheromatous coronary arteries.

## Discussion

Atherosclerosis is a complex disease that brings together inflammation and an oxidative stress environment, where phenotypic switching and migration of VSMC also take part. Therefore, studying the implication of AOC3 in the development of this pathology appears very challenging because this molecule has adhesive and enzymatic functions which are implicated in leukocyte transmigration through endothelium to the inflamed tissue. It is also differentially expressed in VSMC according to their stage of differentiation (3). Our results strongly suggest that under a normal chow diet, the constitutive absence of AOC3 increases the development of atheroma by inducing VSMC dedifferentiation, CD3^+^ lymphocyte, and VSMC recruitment to the plaque in ApoE^−/−^ mice.

Serum AOC3, described as an independent marker of carotid atherosclerosis in diabetic patients (15) and associated with cardiovascular risk factors and early atherosclerotic manifestations (17), was found to increase in mouse serum in the late stage of atherosclerosis. AOC3 could be a marker of the severity of the disease in ApoE^−/−^ mice and in humans (15, 18, 19). Soluble AOC3 is derived mainly from endothelial cells under both physiological and inflammatory conditions, but adipocytes and SMCs also contribute to its production, at least in mice (7, 27). By shedding the membrane-bound AOC3, MMPs could release their soluble form into the plasma in a regulated manner (28, 29). This could explain why in the intima, AOC3 has not increased in 25 weeks old mice whereas AOC3 increased in the abdominal aorta tissues by western blot.

The differences in protocol settings using AOC3 inhibitors could explain the discrepancies regarding AOC3 effects on atherosclerosis (18–21). All these studies (i) used a high fat/high cholesterol diet to accelerate atherogenesis and (ii) used an LDLR^−/−^ mice model, except for two studies, which utilized rabbits (30) and ApoE-/- mice (18). A high-fat cholesterol diet strongly increased biochemical parameters, inflammation, VSMC migration, and proliferation in the atherosclerotic plaque, compared with a chow diet (18, 30). ApoE^−/−^AOC3^−/−^ mice did not present modification of biochemical parameters suggesting that inhibiting AOC3 can be efficient to reduce atherosclerosis by limiting over inflammation induced by the HFD (18–21, 30). Serum AOC3 was correlated with total cholesterol in type 2 diabetic patients (15, 31) and LDL molecules could induce the expression of AOC3 (32). Thus, we preferred to use ApoE^−/−^ model that spontaneously develops atherosclerotic lesions contrary to LDLR^−/−^ mice (33). Moreover, pharmacological AOC3 inhibitors' off-target effects have been highlighted as with SCZ (8, 9) on lysyl oxidase, an enzyme with various implications in the cardiovascular system (22). This observation reinforces the need for studies in knockout models and very specific inhibitors. However, the constitutive deletion of AOC3 is not perfect either, as its total lack during the embryonic development may lead to unknown compensatory mechanisms. AOC3 appears early in the human (34) and mouse (35) embryo, especially in endothelial and VSMC, in a dimeric and enzymatically active form.

Augmentation in the size of atheroma in ApoE^−/−^AOC3^−/−^ mice was characterized by an increased α-SMA at 15 weeks of age when stained by IHC but not by western-blot (Figure 3A). Performed with abdominal lysates, western-blot gives a global α-SMA expression due to VSMC from the lesion and the media layer. α-SMA is a marker of smooth muscle, suggesting enhanced recruitment of VSMC in the plaque. There is no VSMC in the intima of normal rodent vessels (2) contrary to human arteries as shown in Figure 4 (24). This implies dedifferentiation of VSMC from the media which migrate and proliferate into the intima in the absence of AOC3 in mice. Our hypothesis is corroborated by the decrease in α-SMA staining in the media of the aortic sinus associated with a decrease in late differentiation markers such as sm-MHC or smoothelin in the aorta measured by IHC and/or western blot. Sm-MHC is a stringent marker of the smooth muscle lineage (36–38), strictly confined to SMC (36, 38). It is more sensitive to dedifferentiation than α-SMA, which can be expressed in a wide variety of non-derived SMC types within the lesions (37, 39, 40). Even though a secondary effect in response to a mechanism regulated by AOC3 cannot be excluded, the *in vitro* LJP-induced AOC3 inactivation in human aortic VSMC reinforces the existence of a direct effect of AOC3 inactivation on the phenotypical switch of VSMC (Figure 3C), which can increase their migration, collagen synthesis, and proliferation *in vitro* and *in vivo* (20, 21). At least half of VSMC expresses AOC3 in mice and humans but not macrophages in mice. A small part of endothelial cells expresses AOC3, as shown in (18). In humans, CD68 is expressed in the same area as α-SMA and AOC3, suggesting an AOC3 expression by VSMC derived-foam cells. In VSMC, AOC3 does not mediate lymphocyte binding (41, 42) which more likely evokes the implication of mechanisms related to AOC3 enzymatic activity modulation rather than its adhesive properties. The modulations of glucose transport and H_2_0_2_ were previously suggested (6) or demonstrated in other cell types such as adipocytes (12) and chondrocytes (26). Regarding the recent reviews on oxidative stress and atherosclerosis (43–46), the H_2_0_2_ produced by AOC3 might maintain a differentiated VSMC phenotype.

MCP-1/CCL2 is expressed by different cells including VSMC from the media and intimal lesion (2, 47). In addition to its ability to attract in particular T-cells (48), MCP-1 also exhibits a potent chemotactic attraction toward VSMCs (49). Thus, the upregulation of MCP-1/CCL2 at the lesion and the media of 15- and 25-week-old mice aorta, respectively, could participate in the unexpected increase in CD3^+^ T-cells in ApoE^−/−^AOC3^−/−^ lesions and media and the exacerbation of the plaque development.

In conclusion, we characterized, for the first time, this new ApoE^−/−^AOC3^−/−^ mouse model. Unlike in other inflammatory models, beneficial effects of AOC3 deficiency could not be detected when AOC3 was deleted from ApoE^−/−^ mice fed a normal diet. This problem will most likely not exist when atherosclerosis is therapeutically targeted with specific AOC3 inhibitors. The greater mouse atherosclerosis progression could be caused by an abnormal VSMC differentiation without AOC3 and by an indirect increase in T-cell recruitment probably due to an increased expression of MCP-1 in the arterial wall. The mechanisms are not fully identified. Whether modulation of the VSMC phenotypical switch is a direct or indirect effect of AOC3 absence, the question remains to be elucidated in future. The constitutive knock-out model shows some limitations. A conditional and/or tissue-specific knockout of AOC3 would help to discriminate its role in VSMC, but this work rises new perspectives for future research. It emphasizes the need to better understand the involvement of VSMC in human atherogenesis to evolve therapeutic approaches to treat the disease.

## Data availability statement

The original contributions presented in the study are included in the article/Supplementary material, further inquiries can be directed to the corresponding author.

## Ethics statement

The animal study was reviewed and approved by Local Ethics Committee (CELMEA 66) and the Ministry of National Education, Higher Education and Research (Approval number: 01637.02). Written informed consent was not obtained from the individual(s) for the publication of any potentially identifiable images or data included in this article.

## Author contributions

AF, ST, and NM participated in study design, data acquisition, analysis, interpretation, and manuscript preparation. MB, MJ, RB, and CL have contributed to the additional experiments done for the revised version of the manuscript. PL, BF, and SJ participated in the study design and interpretation. All authors attest to each of their substantial contributions to the manuscript, revision, have read, and approved the final version of the manuscript.

## Funding

This work was supported by grants from the Région Lorraine, the Communauté Urbaine du Grand Nancy (CUGN) and investments for the program under grant agreement No ANR-15-RHU-0004.

## Conflict of interest

The authors declare that the research was conducted in the absence of any commercial or financial relationships that could be construed as a potential conflict of interest.

## Publisher's note

All claims expressed in this article are solely those of the authors and do not necessarily represent those of their affiliated organizations, or those of the publisher, the editors and the reviewers. Any product that may be evaluated in this article, or claim that may be made by its manufacturer, is not guaranteed or endorsed by the publisher.
