# Supplementary material for: Increased atherosclerotic plaque in AOC3 knock-out in ApoE−/− mice and characterization of AOC3 in atherosclerotic human coronary arteries
Source: Front Cardiovasc Med. 2022 Sep 13;9:848680. doi: 10.3389/fcvm.2022.848680 (PMC9513161; doi:10.3389/fcvm.2022.848680)
Supplement: Supplementary file 1 [file Data_Sheet_1.docx]

Supplementary Material

# Materials and methods for supplementary Data

**Concentration of soluble VAP-1**

VAP-1 concentrations in the sera were measured by Amine oxidase copper containing 3 (AOC3) kit ELISA (Antibodies online, ABIN810711, Aachen Germany) following the manufacturer’s instructions.

**Pro-inflammatory markers and VSMC differentiation markers in abdominal aortas**

The presence of pro-inflammatory markers in abdominal aortas was measured using RayBio Mouse Atherosclerosis Antibody Array C (RayBiotech, Norcross, Georgia, USA, AAM-ATH-1). The array consisted of 22 different soluble signalling factor antibodies (listed in supplementary figure 4) spotted in duplicate onto a PVDF membrane. Briefly, the abdominal aortas were individually homogenised in 200 µl buffer given by the manufacturer complemented with a protease inhibitor cocktail (Roche, Mannheim, Germany). Protein concentration was measured with DC™ Protein Assay (BioRad, 5000116). These homogenates were used both for western blot (see paragraph 2.4) and a Mouse Atherosclerosis Antibody Array C. Equal amounts of protein from 7 tissue lysate samples per group were pooled to reach 250 µg of protein. The following procedure was performed according to the manufacturer’s instructions. Chemiluminescence detection was executed with an LAS 4000 analyser system (Fujifilm Life science, Stamford, USA). The signal intensity of each spot was quantified by Multi Gauge V3 (Fujifilm, Life science, Stamford, USA). The signal intensity of the negative control was subtracted from each spot and the values were normalized to the mean of positive controls. The results were expressed in Arbitrary Units.

The previous individual lysates from abdominal mouse aortas were denatured and put in a SDS/PAGE gel a Mini-PROTEAN Tetra Cell (BioRad, Marnes-La-Coquette, France) and transferred onto a nitrocellulose membrane (BioRad, Marnes-La-Coquette, France) with a Mini Trans-Blot Module. Membranes were blocked with 5% non-fat dry milk in 0.1% Tween 20 in TBS buffer (10 mM Tris-Base and 15 mM NaCl, pH 7.5) for 1h at room temperature. The membranes were blotted with the following primary antibodies directed against: anti-smooth muscle myosin heavy chain SM-MHC (BioMedical Technologies, BT-562, Stoughton, USA), anti-alpha smooth muscle actin (α−SMA) (Sigma A5228, Merck KGaA, Darmstadt, Germany), anti h-caldesmon (Santa Cruz, sc15374, Dallas, TX, USA), anti-smoothelin (Santa Cruz, sc28562, Dallas, TX, USA) and GAPDH (Santa Cruz, sc25778, Dallas, TX, USA) in the blocking solution. After several washes, the membrane was incubated with an appropriate HRP-coupled secondary antibody in the blocking solution for 1 h. Following washes, the protein bands were visualized using the Chemiluminescence Kit (Immun-Star™ WesternC™ Chemiluminescence Kit, Bio-Rad, Marnes-La-Coquette, France) on a Fujifilm Luminescent Image Analyzer LAS4000 System.

**Confocal imaging and co-localisation**

For mice, 10 µm OCT aortic sinus sections from ApoE^-/-^ mice at 15 and 25 weeks of age were fixed in PAF 4% for 15 min. Permeabilization was performed with TBS-T0.05% containing triton 0.1% for 10 min. Slides were blocked in TBS-T0.05% containing BSA 1% and donkey serum 5% for 45 min. Slides were incubated at the same time with 2 primary antibodies diluted in the blockage solution (rabbit anti-AOC3 (1:200, from S. Jalkanen) either with a mouse cyanine-conjugated anti α-SMA antibody (1:200, Sigma C6198 by Merck, Darmstadt, Germany), with a rat anti-CD31 (1:50, BD Pharmingen, 550274, Le Pont de Claix, France), or with a rat anti-MOMA-2 (1:50, MAB1852, Merck, Darmstadt, Germany) for 1h at room temperature, excepted for the negative control. After several washes, sections were incubated with fluorescent secondary antibodies at dilution 1:200, all purchased from Invitrogen (by Thermo Fisher Scientific, Illkirch, France): Alexa fluor 488 donkey anti-rabbit (A21206) alone or an Alexa fluor 555 goat anti-rabbit (A21429) with an Alexa fluor 488 donkey anti-Rat (A21208) for 1h, in the dark, at room temperature. Slides were washed and incubated with DAPI (4', 6-diamidino-2-phenylindole, 1:20000, Thermo Fisher Scientific, 10184322, Illkirch, France) for 15 min at room temperature, in the dark. Cover slide were fixed with an aqueous mounting medium (Sigma, F4680, by Merck, Darmstadt, Germany).

For human, 5 µm paraffin human coronary sections from healthy, fatty streak and atheroma areas were deparaffinized. Antigenic sites were unmasked with citrate 10 mM, pH6 for 30 min at 65°C under agitation. Section were incubated with TBS-T0.05% containing Triton 0.1% for permeabilization. Unspecific sites were blocked in TBS-T0.05%/BSA 1% /donkey serum 5% for 45 min. Slides were incubated with 2 primary antibodies at the same time, diluted in the blockage solution: a rabbit anti-AOC3 (S. Jalkanen 1:200) with a mouse anti α-SMA antibody at (1:100, Sigma A5228, by Merck, Darmstadt, Germany), for 1h at room temperature, excepted for the negative control. After washing, fluorescent secondary antibodies (dilution 1:200, Invitrogen by Thermo Fisher Scientific, Illkirch, France), Alexa fluor 488 donkey anti-Rabbit (A21206) and an Alexa fluor 555 donkey anti-mouse (A21127) were mixed and incubated on the section for 1h in the dark, at room temperature. Slides were washed and incubated with DAPI (4', 6-diamidino-2-phenylindole, 1:20000, Thermo Fisher Scientific, 10184322, Illkirch, France) for 15 min at room temperature. Cover slide were fixed with an aqueous mounting medium (Sigma, F4680, by Merck, Darmstadt, Germany).

Mouse and human immuno-stained tissue sections were imaged by confocal microscopy with the objective Plan Apo 60× (Nikon C2). To show the presence and localization of the protein AOC3 in endothelial cells (CD31 or VWF), vSMC (α-SMA) and macrophages (MOMA-2), two different methods have been applied: plot profile of pixel intensity and Manders coefficient calculus. First, we displayed a two dimensional-graph of the intensity of pixels along a line within the image using the ‘Plot Profile’ plugin of ImagJ. This pixel profile plot shows the localization of proteins within the cell. The ‘JACoP’ plugin of ImageJ calculates the Manders coefficients in a selected region within an image. The Manders coefficients (M1 and M2) are co-occurrence measures that calculate the percentage of total signal from one channel which overlaps with the signal from the other. Here M1 is the percentage of signal from the green channel overlapping with the signal from the red channel. M2 is the percentage of signal from the red channel overlapping with the signal from the green channel. 2 to 4 areas per slide were analyzed in 3 to 4 animal or human samples per group.

**Spleen lysates for RT- and real time PCR**

Pieces of mouse spleen were put in 1 ml of Isol-RNA Lysis Reagent (5 Prime, VWR International, Fontenay-sous-Bois, France) and crushed in the tissue-lyser for 15 min. RNA spleen was performed with RNeasy kit (Qiagen, ID: 74104, Courtaboeuf Cedex, France) according to the manufacturer’s instructions. The RNA concentration and quality were assessed by using a NanoDrop® ND-1000 UV-Vis Spectrophotometer (Thermo Scientific, Villebon sur Yvette, France). The cDNAs were synthesized from 1 µg of total RNA with iScript^TM^ advanced cDNA synthesis Kit (Bio-Rad, 5000116, Marnes-La-Coquette, France). Real-time PCR analyses were performed by incubating 12.5 µl of SYBR Green PCR Master Mix (Bio-Rad), 300 nM of sense and anti-sense primer (supplementary table 1) and 250 ng of cDNA in a total reaction volume of 25 µl. RT-qPCR was carried out using an iCyler iQ thermal cycler (MyiQ Single-Color Real-Time PCR Detection System, Bio-Rad,) according to the manufacturer’s instructions. The gene encoding GAPDH was used as a standard. Each sample was tested in duplicate. The 2^-ΔΔCt^ methods were used for calculation.

# Supplementary Tables

Supplementary table 1. Antibody details used in IHC for mouse and human samples.

| **Antibodies for IHC:** | **Reference product** | **Supplier, city, country** |
| --- | --- | --- |
| **-on mouse tissues** |  |  |
| monoclonal rat anti-mouse macrophage (MOMA-2) | MAB1852 | Merck, Darmstadt, Germany |
| a rabbit polyclonal anti-CD3 | A0452 | Dako, Glostrup, Denmark |
| rabbit polyclonal anti-alpha smooth muscle actin (α-SMA) | PA1-37023 | Thermo Fisher Scientific, Rockford, USA |
| rabbit anti-smooth muscle myosin heavy chain SM-MHC | BT-562 | BioMedical Technologies, Stoughton, USA |
| Rabbit polyclonal anti-AOC3 |  | Made by S. Jalkanen |
| **-on human tissues** |  |  |
| Rabbit anti-human CD68 | ab213363 | 1:8000, Abcam, Cambridge, UK |
| mouse anti-AOC3 | sc373924 (E10) | Santa Cruz, , Heidelberg, Germany |
| a rabbit anti-human Von Willbrand Factor (VWF) | A-0082 | Dako, Glostrup, Denmark |
| mouse anti α-SMA | A5228 | Sigma, by Merck, Darmstadt, Germany |
| **Secondary antibodies for mouse and human samples** |  |  |
| Immpress Excel rabbit IGG | MP-7601 | Eurobio scientific, Courtaboeuf, France |
| Immpress Excel mouse IGG | MP7602 | Eurobio scientific Courtaboeuf, France |

**Supplementary table 2. Primer sequences used for RT-qPCR mouse cDNA analysis.**

The forward and reverse sequences of primers are given with the product length (in base pair, bp), the hybridization temperature (in °C) and the expected product length.

| **Primers for:**  **Mouse** |  | **Sequences 5’ – 3’** | **product length**  **(bp)** | **T_m_ (°C)** |
| --- | --- | --- | --- | --- |
| GAPDH | For | AACTTTGGCATTGTGGAAGG | 132 | 60 |
|  | Rev | GGATGCAGGGATGATGTTCT |  |  |
| TNFα | For | GTAGCCCACGTCGTAGCAAAC | 118 | 60 |
|  | Rev | CTGGCACCACTAGTTGGTTGTC |  |  |
| IFNγ | For | GCCACGGCACAGTCATTGAAA | 261 | 60 |
|  | Rev | TCGCCTTGCTGTTGCTGAAGA |  |  |
| TGFβ | For | AGCAACAATTCCTGGCGTTACCTT | 221 | 60 |
|  | Rev | TGGGCTCGTGGATCCACTTC |  |  |
| IL-10 | For | GGCGCTGTCATCGATTTCT | 111 | 60 |
|  | Rev | ATTCATTCATGGCCTTGTAGACAC |  |  |
| **Human** |  |  |  |  |
| RPS29 | For  Rev | AGATGGGTCACCAGCAGCTCTACTG  AGACGCGGCAAGAGCGAGAA | 72 | 64 |
| AOC3 | For  Rev | CTGGTTTGGCCTCTACTACAACA  CAAGGGCCTTGTGGTTCACT | 89 | 64 |
| α-SMA | For  Rev | ACTGCCTTGGTGTGTGACAATGG  TGGTGCCAGATCTTTTCCATG | 248 | 62 |
| h-Caldesmon | For  Rev | GTCACCAAGTCCTACCAGAAGA  GCTGCTTGATGGGTCGATTTGA | 821 | 62 |
| Smoothelin | For  Rev | GGCAGTGTCACTCACGTCAC  CTGATCCAGCATCTTGTCCA | 231 | 62 |

**Supplementary table 3: General parameters of ApoE^-/-^ and ApoE^-/-^SSAO^-/-^ mice**.

Number of female mice per groups is indicated in function of the genotype and the age. Total cholesterol, LDL-cholesterol, and triglyceride concentrations are given in mmol/l. The Mann Whitney test gave non-significant differences (ns).

| Age | 15 weeks | | 25 weeks | | Statistics |
| --- | --- | --- | --- | --- | --- |
| Genotype | ApoE^-/-^ | ApoE^-/-^VAP^-/-^ | ApoE^-/-^ | ApoE^-/-^VAP^-/-^ | ns |
| n | 7 | 7 | 6 | 7 | ns |
| Total cholesterol (mmol/l) | 9.4+/-2.2 | 11+/-2.2 | 10.1+/-2.5 | 9.1+/-1.1 | ns |
| LDL-cholesterol (mmol/l) | 3.8+/-0.8 | 3.8+/-0.6 | 3.6+/-0.7 | 3.7+/-1.1 | ns |
| Triglyceride (mmol/l) | 0.7+/-0.2 | 0.8+/-0.2 | 0.7+/-0.1 | 0.5+/-0.2 | ns |
| Body weight (g) | 19.0+/-1.2 | 20.6+/-2.2 | 22.4+/-3.8 | 20+/-3.2 | ns |

# Legend to supplementary figures

**Supplementary Figure 1 : AOC3 during atherosclerosis in ApoE^-/-^ mice**

The concentration of soluble AOC3 was measured by ELISA in serum from 8, 9 ,10 and 5 ApoE^-/-^ mice at 11, 15, 25 and 70 weeks of age respectively **(A)**; and in 8, 5 and 3 mice at 70 weeks of age, from WT, ApoE^-/-^ and VAP-1^-/-^ mice, respectively **(B)**. Results are expressed in ng/ml. **(C)** AOC3 protein expression in abdominal aortas from ApoE^-/-^ mice were measured by western blot and results **(D)** are given as the relative expression normalized by ponceau signal. At 11 weeks, n=4, at 15 weeks, n=7, at 25 weeks, n=6 and at 70 weeks, n=4. Each dot represents the mean of duplicate for an animal and the lines represent the mean +/- SD. **(E)** AOC3 protein expression in aortic sinus from 15- and 25-week-old ApoE^-/-^ mice was measured by immunohistochemistry. Representative pictures are provided at objective x10 and x40. The scale bar represents 100 µm. **(F)** The AOC3 signal in the media and the lesion are expressed in % of the area measured. For all graphs, *, p<0.05. Each dot stands for a single animal and the line represents the mean of the group +/- SD.

**Supplementary figure 2: Lesion size in aortic sinus of female mice**

Lesion size was quantified after oil red O staining in the aortic sinus of female 15- and 25-week-old ApoE^-/-^ and ApoE^-/-^AOC3^-/-^ mice. (n-6-9/group). (A) Representative micrographs of mouse aortic sinus for each group. The scale bar = 100 µm. (B) Plaque surface quantification expressed as µm^2^. Each dot is the result of one animal and the line represents the mean of the group +/- SD. *, p<0.05.

**Supplementary figure 3: Lesion size in the thoracic Aorta in males**

Lesion size after oil red O staining was quantified in the male thoracic aorta of 15- and 25-week-old ApoE^-/-^ and ApoE^-/-^AOC3^-/-^ mice (n-6-9/group). **(A)** Representative micrograph of partial thoracic, arch and descending aorta for each group. Bar scale represents 1 mm. **(B)** the lesion size quantification is expressed in % of the aorta surface. **(C)** Plasma total cholesterol is given in mmol/l. Each dot is the result of one animal and the line represents the mean of the group +/- SD. *, p<0.05. **(D)** abdominal segment of ApoE^-/-^ and **(E)** ApoE^-/-^AOC3^-/-^ mice at 25 weeks of age were presented to illustrate the presence of red atherosclerotic lesions at this sight, considering the associated assessment of pro-inflammatory and VSMC differentiation markers. The black arrows indicate the iliac bifurcation. The space between 2 graduations measure 1 mm.

**Supplementary figure 4: Cytokine expressions in the spleen and the abdominal aorta**.

**(A)** IL10, **(B)** TGFβ, **(C)** IFNγ and **(D)** TNFα mRNAs were measured by quantitative RT-PCR in the female ApoE^-/-^ (●) and ApoE^-/-^AOC3^-/-^ (○) spleens from 15- and 25-week-old female mice. GAPDH mRNA was used for normalization. In graphs, the individual values for each animal and the average values are presented as a horizontal line for ApoE^-/-^ and ApoE^-/-^AOC3^-/-^ mice. Each sample was performed as duplicate. No statistical difference was found. A mouse atherosclerosis-associated cytokines array was performed with abdominal aortas from ApoE^-/-^ and ApoE^-/-^AOC3^-/-^ female mice. Blots of the protein array are presented in **(E)** and the quantification of the relative signal intensity is shown in **(F)** for mice at 15 weeks of age. The blots **(G)** and their quantifications **(H)** are given for mice at 25 weeks of age. The map of the different cytokines is shown in **(I)**. Fibroblast growth factor-basic (bFGF), CD40 (TNFRSF5), eotaxin-1 (CCL11), granulocyte colony-stimulating factor (G-CSF), granulocyte-macrophage colony-stimulating factor (GM-CSF), IFN-γ, interleukin (IL)-1α, IL-1β, IL-2, IL-3, IL-4, IL-5, IL-6, IL-13, L-selectin (CD62L), monocyte chemoattractant protein-1 (MCP-1)(CCL2), macrophage colony-stimulating factor (M-CSF), macrophage inflammatory protein-3α (MIP-3α) (CCL20), P-selectin, RANTES (CCL5), tumour necrosis factor-α (TNF-α), and vascular endothelium growth factor-A (VEGF-A). Quantification of the relative signal intensity is given for 15-week-old mice **(F)** and for 25-week-old mice in **(H)** in arbitrary units. Results are duplicates from a pool of 7 abdominal aortas for each group.

**Supplementary figure 5: AOC3 and α-SMA, CD31 and MOMA-2 co-localization in the aortic sinus from ApoE^-/-^ mice.**

Representative pictures of **(A)** AOC3 and α-SMA, **(B)** AOC3 (red) and CD31 (green) and **(C)** AOC3 (red) and MOMA-2 (green) co-immunofluorescent stainings observed by confocal microscopy are presented with their plot profile of pixel intensity and M1 and M2 Manders coefficient calculus when it was possible. This show if there is colocalization between AOC3 and VSMC, endothelial cells or macrophages in the media and the intima from 15- and 25-week-old ApoE^-/-^ mice. Line scans show the distribution of fluorescence signals along the yellow line within the indicated cell in the magnified image. The white dotted line separate intima (i) and media (m) layers. Bleu arrows indicate the endothelium. M1 and M2 are the Manders coefficient calculated in the selected zone (dotted line) in the 60x images. Lumen (lu), plaque (pl), blood vessel (ve), adventitia (a), adipocytes (ad) are sometime indicated on pictures. Scale bars represent 10 µM at 20x, 5 µm at 60x and 2 µm for the magnification.

**Supplementary figure 6: Co-immnunofluorescence in human healthy, fatty streak and Atheroma coronary arteries**

Representative pictures of AOC3 (red) and α-SMA (green) in healthy **(A)**, fatty streak **(B)** and atheroma **(C)** after co-immunofluorescent staining observed by confocal microscopy are presented with their plot profile of pixel intensity and M1 and M2 Manders coefficient calculus when it was possible. This show if there is co-localization between AOC3 and VSMC in the media (m) and intima (i). Line scans show the distribution of fluorescence signals along the line within the indicated cell in the magnified image. The white dotted line separate intima (i) and media (m) layers. Bleu arrows indicate the endothelium. M1 and M2 are the Manders coefficient calculated in the selected zone (yellow thin dotted line) in the 60x images. Lumen (lu) and adventitia (a) are indicated on the 20x pictures. Scale bars represent 10 µM at 20x, 5 µm at 60x and 2 µm for the magnification.
