# Supplementary figures and images for: Increased atherosclerotic plaque in AOC3 knock-out in ApoE−/− mice and characterization of AOC3 in atherosclerotic human coronary arteries
Source: Front Cardiovasc Med. 2022 Sep 13;9:848680. doi: 10.3389/fcvm.2022.848680 (PMC9513161; doi:10.3389/fcvm.2022.848680)

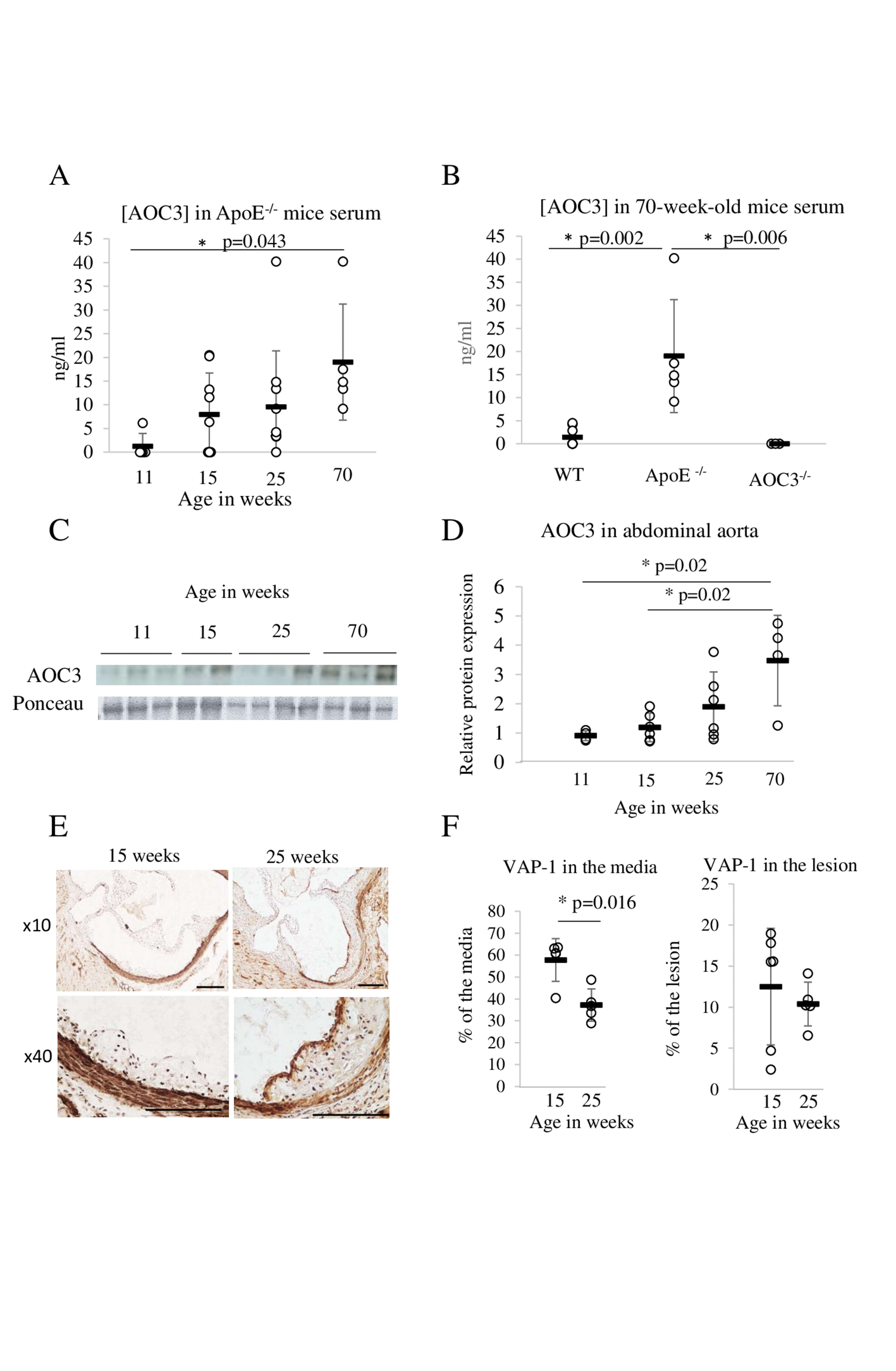

Supplement: Supplementary file 2 [file Image_1.TIFF]

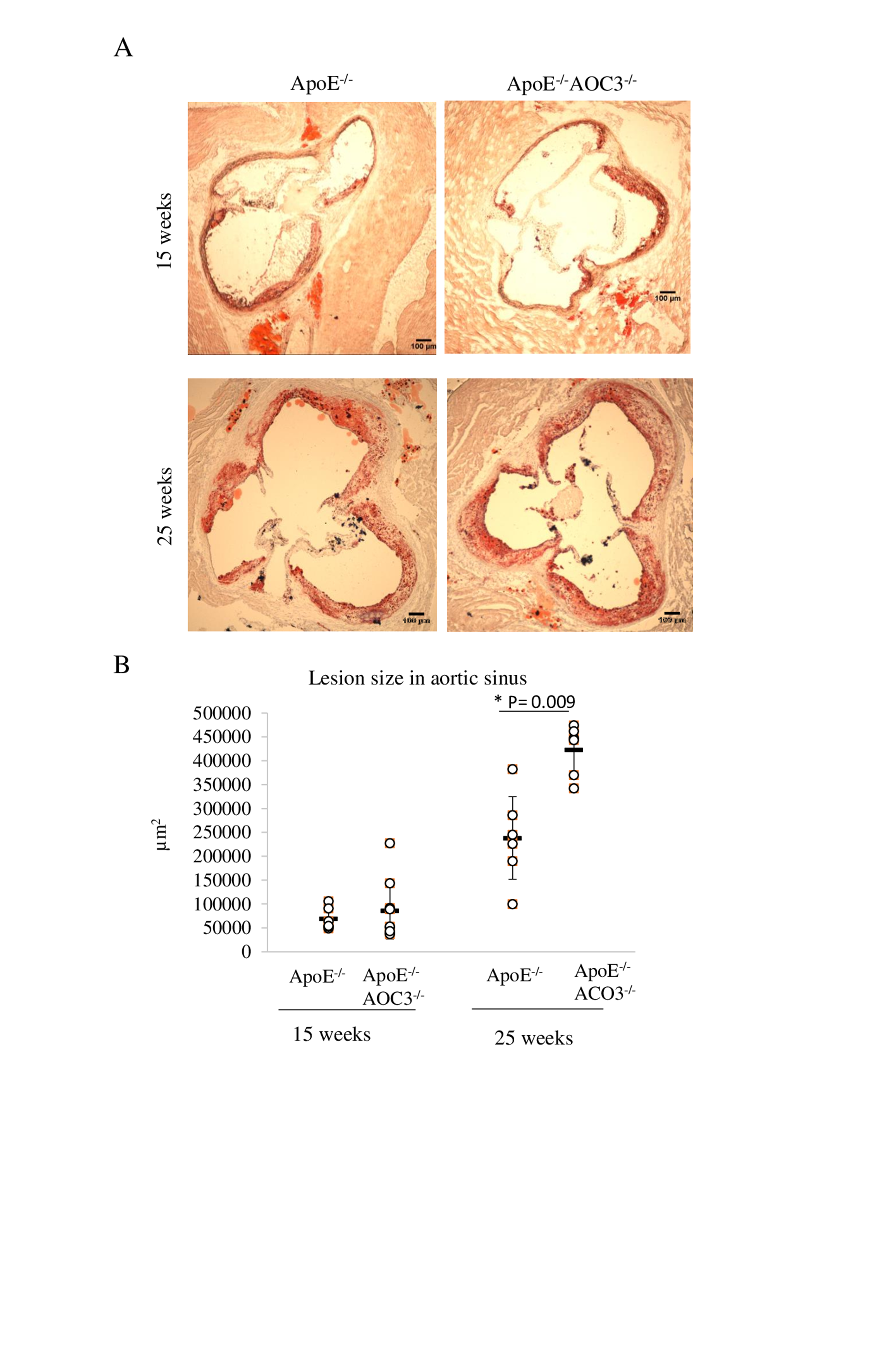

Supplement: Supplementary file 3 [file Image_2.TIFF]

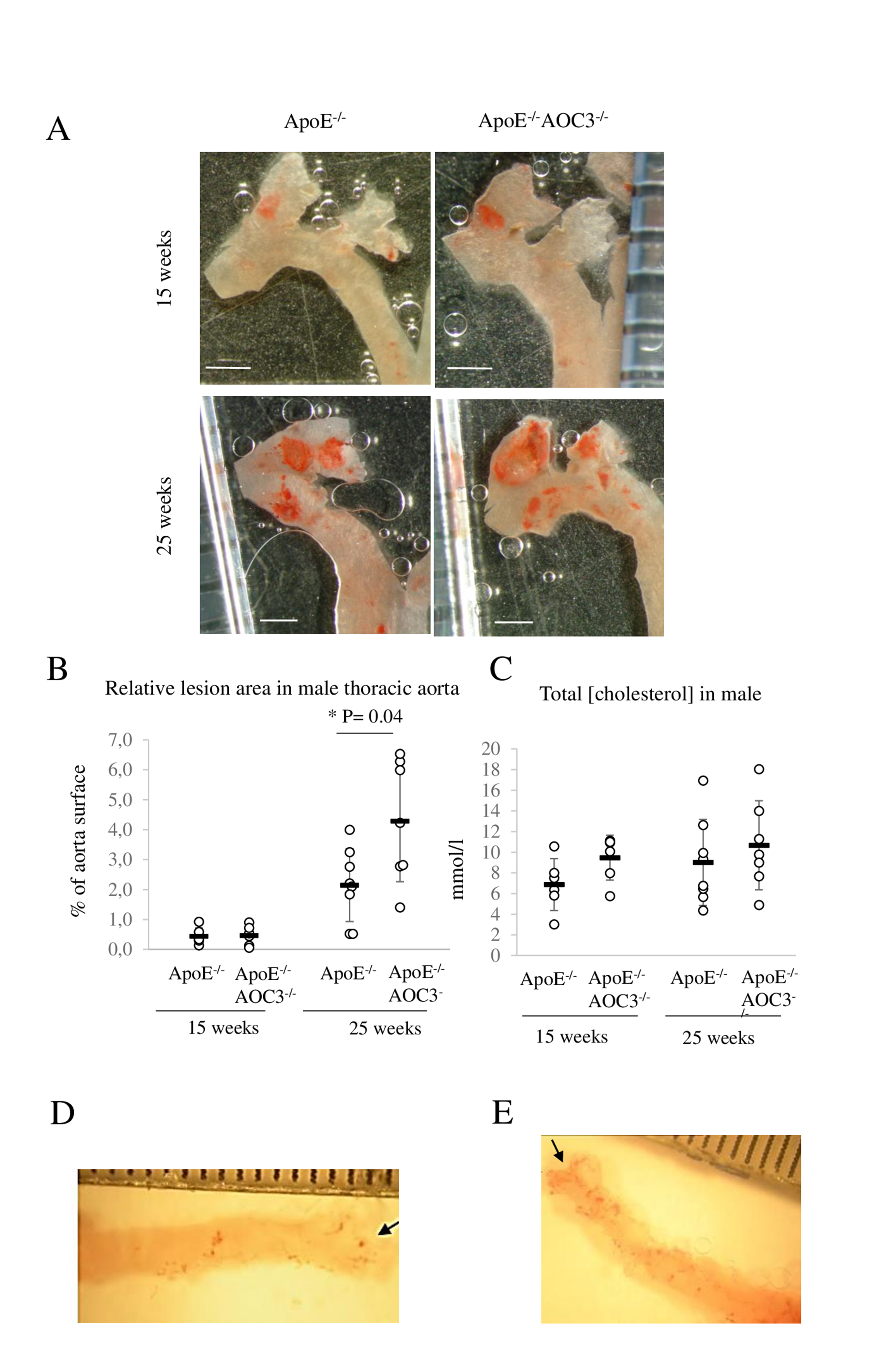

Supplement: Supplementary file 4 [file Image_3.TIFF]

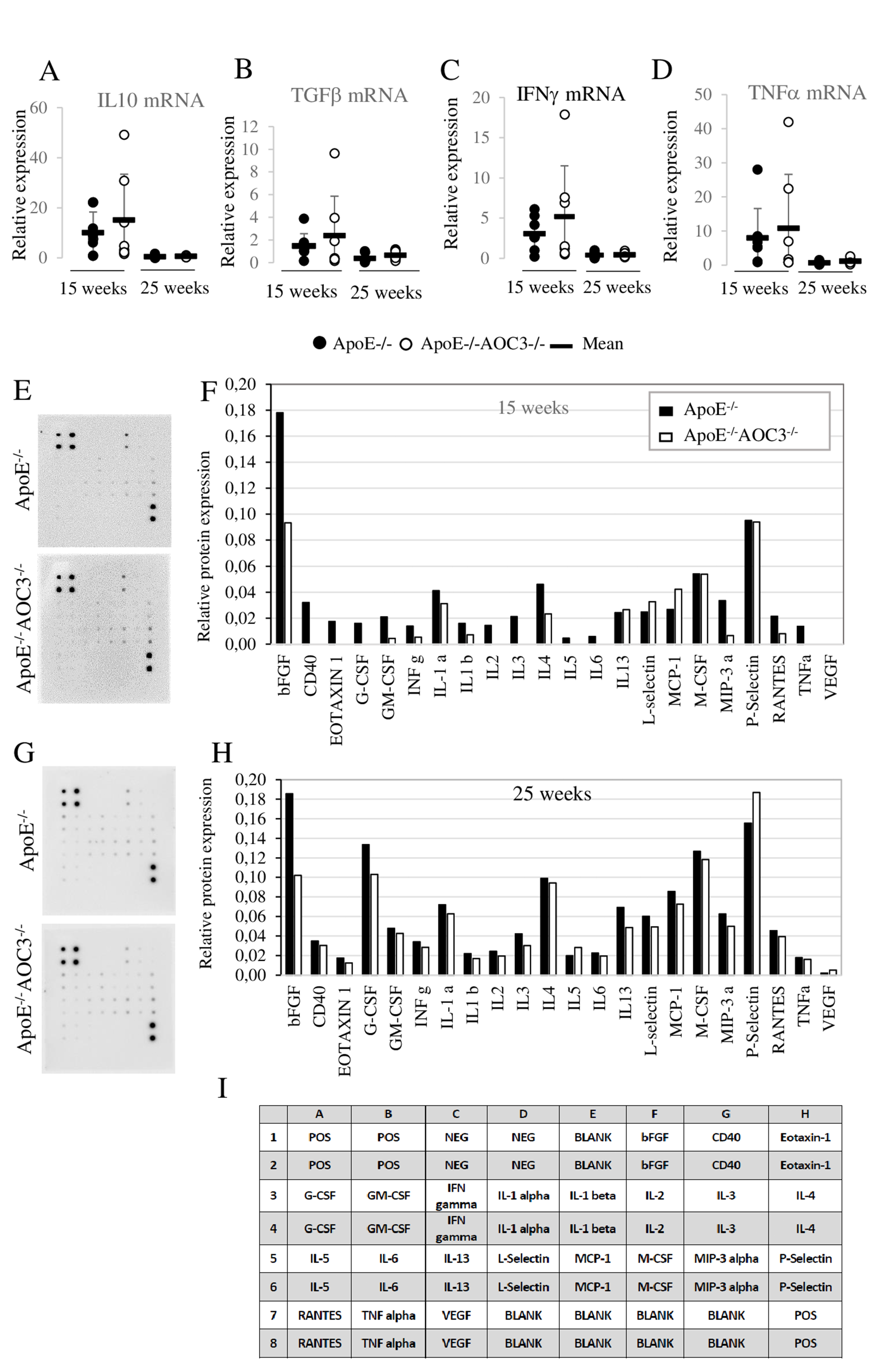

Supplement: Supplementary file 5 [file Image_4.TIFF]

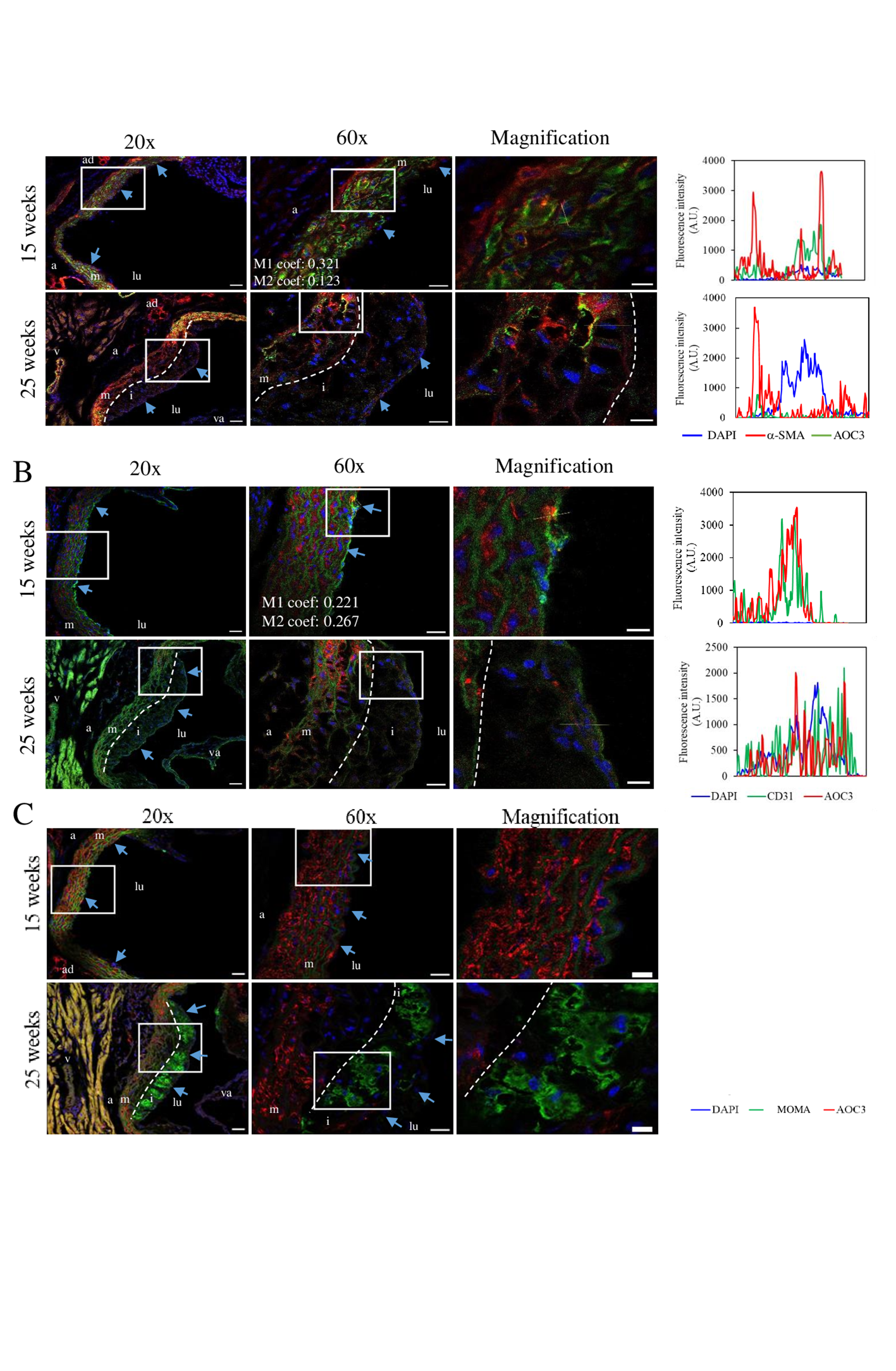

Supplement: Supplementary file 6 [file Image_5.tiff]

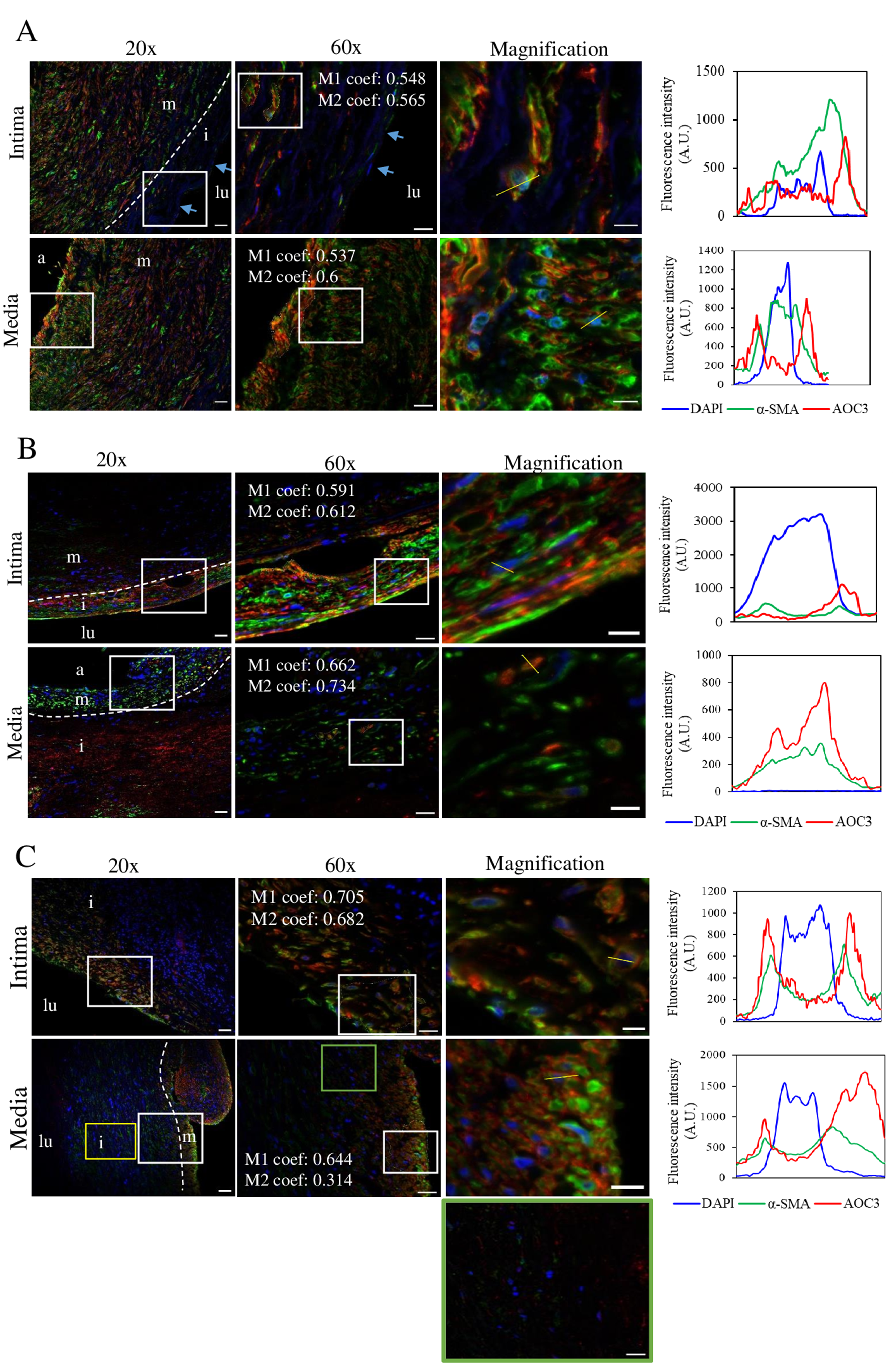

Supplement: Supplementary file 7 [file Image_6.tiff]
